# Enhanced Antioxidative Defense by Vitamins C and E Consumption Prevents 7-Day High-Salt Diet-Induced Microvascular Endothelial Function Impairment in Young Healthy Individuals

**DOI:** 10.3390/jcm9030843

**Published:** 2020-03-20

**Authors:** Lidija Barić, Ines Drenjančević, Martina Mihalj, Anita Matić, Marko Stupin, Luka Kolar, Zrinka Mihaljević, Ines Mrakovčić-Šutić, Vatroslav Šerić, Ana Stupin

**Affiliations:** 1 Department of Physiology and Immunology, Faculty of Medicine Josip Juraj Strossmayer University of Osijek, J. Huttlera 4, Hr-31000 Osijek, Croatia; rasiclid@gmail.com (L.B.); ines.drenjancevic@mefos.hr (I.D.); martina.mihalj@gmail.com (M.M.); anita.matic@mefos.hr (A.M.); marko.stupin@gmail.com (M.S.); lukakolar.vu@gmail.com (L.K.); zrinka.mihaljevic@mefos.hr (Z.M.); 2 Scientific Center of Excellence for Personalized Health Care, Josip Juraj Strossmayer University of Osijek, Trg Svetog Trojstva 3, Hr-31000 Osijek, Croatia; 3 Department of Dermatology and Venereology, Osijek University Hospital, J. Huttlera 4, HR-31000 Osijek, Croatia; 4 Department for Cardiovascular Disease, Osijek University Hospital, J. Huttlera 4, HR-31000 Osijek, Croatia; 5 Department of Physiology and Immunology, Medical Faculty University of Rijeka, Ul. Braće Branchetta 20/1, HR-51000 Rijeka, Croatia; ines.mrakovcic.sutic@medri.uniri.hr; 6 Department of Clinical Laboratory Diagnostics, Osijek University Hospital, J. Huttlera 4, HR-31000 Osijek, Croatia; seric.vatroslav@kbo.hr; 7 Department of Pathophysiology, Physiology and Immunology, Faculty of Dental Medicine and Health Josip Juraj Strossmayer University of Osijek, Cara Hadrijana 10E, HR-31000 Osijek, Croatia

**Keywords:** high-salt diet, microcirculation, endothelium, oxidative stress, antioxidative defense, vitamin C, vitamin E, renin-angiotensin system

## Abstract

This study aimed to examine whether the oral supplementation of vitamins C and E during a seven-day high salt diet (HS; ~14 g salt/day) prevents microvascular endothelial function impairment and changes oxidative status caused by HS diet in 51 (26 women and 25 men) young healthy individuals. Laser Doppler flowmetry measurements demonstrated that skin post-occlusive reactive hyperemia (PORH), and acetylcholine-induced dilation (AChID) were significantly impaired in the HS group, but not in HS+C+E group, while sodium nitroprusside-induced dilation remained unaffected by treatments. Serum oxidative stress markers: Thiobarbituric acid reactive substances (TBARS), 8-iso prostaglandin-F2α, and leukocytes’ intracellular hydrogen peroxide (H_2_O_2_) production were significantly increased, while ferric-reducing ability of plasma (FRAP) and catalase concentrations were decreased in the HS group. All these parameters remained unaffected by vitamins supplementation. Matrix metalloproteinase 9, antioxidant enzymes Cu/Zn SOD and glutathione peroxidase 1, and leukocytes’ intracellular superoxide production remained unchanged after the protocols in both HS and HS+C+E groups. Importantly, multiple regression analysis revealed that FRAP was the most powerful predictor of AChID, while PORH was strongly predicted by both FRAP and renin-angiotensin system activity. Hereby, we demonstrated that oxidative dis-balance has the pivotal role in HS diet-induced impairment of endothelial and microvascular function in healthy individuals which could be prevented by antioxidative vitamins consumption.

## 1. Introduction

Numerous studies have confirmed the strong positive correlation between high-salt (HS) intake and elevated blood pressure (BP) levels, which consequently leads to the development and progression of arterial hypertension and other cardiovascular (CV) diseases [1,2,3]. On the other hand, in the last few decades it became evident that the adverse effects of HS intake can also be independent of changes in BP, e.g., results of the Intersalt study reported a stronger correlation between HS intake and cerebrovascular insult than between BP and cerebrovascular insult [4,5]. Similarly, it was demonstrated that HS intake is associated with hypertensive total organ damage independently of BP level [6]. A growing body of evidence indicates that HS intake is highly detrimental to vascular and endothelial function, even in the absence of arterial BP changes. Endothelial dysfunction presents an early (and still reversible) pathological event in the development of CV diseases, and it seems that impaired microvascular reactivity to various stimuli may be the first sign of the development of endothelial dysfunction [7]. 

It has been demonstrated that the HS diet (only one HS meal and seven-day HS diet) impairs flow-mediated dilation (FMD) of the brachial artery, independently of changes in BP in healthy individuals [8,9,10,11]. However, one HS diet meal did not affect the postprandial reactive hyperemia index measured by reactive hyperemia peripheral arterial tonometry (RH-PAT) (microvascular function) in healthy individuals [11]. Therefore, in order to study the effect of HS on microvascular function, as well as to study the potential mechanisms that mediate such effect, the HS diet used in studies in healthy individuals usually provided 250 to 400 mmol of Na+/day (14.6 to 23.4 g of NaCl/day), and in most studies, the diet was lasted from 5 to 7 days [12]. Regarding microcirculation, forearm skin microvascular reactivity in response to vascular occlusion [13], acetylcholine (ACh) stimulation [14] and local heating (measured by laser Doppler flowmetry) was attenuated by a seven-day HS diet in healthy individuals [15], in the absence of BP changes, or changes in body composition and body fluid status [14]. 

Potential mechanisms that mediate such BP-independent HS diet-induced impairment of vascular and endothelial function include a decrease in nitric oxide (NO) production and endothelium-dependent dilation by increasing the level of oxidative stress [16,17]. The decrease in NO bioavailability is probably a result of oxidation of endothelium-derived NO or other cofactors essential for NO synthesis [17,18]. Besides NO, HS diet-induced impairment of vascular (endothelial) function may involve other endothelial vasoactive pathways including vasodilator and/or vasoconstrictor metabolites of arachidonic acid. Cavka et al. demonstrated that HS diet-induced impairment of microvascular response to vascular occlusion was accompanied by an increase of serum protein concentration of thromboxane (TXA2), a vasoconstrictor metabolite of arachidonic acid derived by cyclooxygenase enzymes (COX-1 and/or COX-2) [13]. The same research group reported that endothelium-dependent vasodilation of arterioles isolated from gluteal subcutaneous fat following a seven-day HS diet was no longer NO-dependent, but that COX and cytochrome P450 (CYP450) vasodilator mediators took part in this vasodilation [8]. Furthermore, HS diet leads to reduced expression and activity of antioxidant enzymes, which alone or together with increased activity of prooxidant enzymes contributes to increased oxidative stress level in experimental animals [12,16,19]. Moreover, certain studies have reported that in vitro administration of antioxidants, such as superoxide dismutase (SOD) or SOD mimetic TEMPOL, a superoxide scavenger, reverses oxidative status, and restores endothelium-dependent dilation to levels measured in animals on normal salt diet [16,20]. Recent research efforts of both animal and clinical studies have focused on antioxidant-based therapeutics to alleviate the oxidative stress involved in the pathophysiology of CV diseases [21]. Despite uncertainty regarding the health benefits, data show that a number of adults have taken or are taking vitamin supplements with antioxidant properties (12.7% and 12.4% of US adults took vitamin E and C supplements, respectively; around 20% of adults in Germany took dietary supplements containing vitamin C and E) [22,23,24]. However, there is a lack of studies examining the effects of administration of antioxidants, such as vitamins on microvascular function during HS loading in humans. For example, only one study by Greaney et al. reported that localized microdialysis of vitamin C, considered as non-specific scavenger of the reactive oxygen species (ROS), has completely restored the NO-dependent component of arteriolar dilation in response to local heating in humans on an HS diet [15].

Matrix metalloproteinase 9 (MMP-9) is an enzyme that actively participates in remodeling of blood vessels due to oxidative and vascular nitrosative stress by matrix degradation [25]. Hypertensive patients express higher levels of MMP-9 [26], and MMP-9 activity is enlarged in arteries under high pressure compared to vessels under normal pressure [27]. Still, there is a paucity of data on the activity of MMP-9 and its role in vascular remodeling after short-term HS intake in young healthy individuals. 

Hence, the aim of this study was to test the effect of a seven-day HS diet on microvascular endothelial function and biochemical parameters of oxidative stress level (e.g., thiobarbituric acid reactive substances (TBARS), ferric-reducing ability of plasma (FRAP), intracellular ROS production, 8-iso prostaglandin F2α (8-iso-PGF2a), antioxidative enzymes), and to examine whether the oral supplementation of non-specific ROS scavengers (vitamin C and vitamin E) during seven-day HS loading will change oxidative status and potentially prevent microvascular endothelial function impairment in otherwise healthy normotensive individuals. The aim was also to investigate whether a short-term HS diet lasting for 7 days potentially induces changes in serum MMP-9 levels, thus preparing the substrate for vascular remodeling.

## 2. Experimental Section

### 2.1. Study Population

A total of 51 young healthy subjects (26 women and 25 men), age ranged from 18–24 years took part in this study. Written informed consent was obtained from each subject. The study protocol and procedures conformed with the standards set by the latest revision of the Declaration of Helsinki and were approved by the Ethical Committee of the Faculty of Medicine, University of Osijek (Cl: 602-04/15-08/08; No: 2158-61-07-15-68).

The study was performed in the Laboratory for Clinical and Sport Physiology, Department of Physiology and Immunology at Faculty of Medicine, University of Osijek. All the examinations were performed in the morning after an overnight fasting. Exclusion criteria were history of hypertension, coronary artery disease, diabetes, hyperlipidemia, renal impairment, cerebrovascular and peripheral artery disease. Obese individuals (body mass index (BMI) > 30 kg/m^2^), or individuals taking oral contraceptives, antihypertensive agents, anti-inflammatory non-steroidal drugs, steroids, or other drugs that could affect the endothelium were also excluded from the study. Women entered the study protocol in the different phases of the menstrual cycle (randomized) in order to eliminate the effect of sex hormones fluctuation during the menstrual cycle.

### 2.2. Study Protocol

Subjects were divided into two groups: the HS group and the HS+C+E group. Initial characteristics of participants during selection to study are shown in Table 1. At the beginning of the protocol, all subjects of both groups were instructed to maintain a low-salt (LS) diet for 7 days, with an intake of less than 3.5 g of salt per day (DASH eating plan; US Department of Health and Human Services, 2006), which was considered a “wash-out” period. Then, subjects in HS group took 7-day HS diet protocol which implied intake of 3.5 g of salt per day in diet according to the DASH diet and additional 11.7 g of salt per day supplemented in a form of a salt powder. Participants in HS+C+E group have taken the same 7-day HS diet protocol supplemented with simultaneous per oral antioxidative vitamins intake, 1000 mg of vitamin C per day and 800 IU of vitamin E per day. 

### 2.3. Salt Sensitive/Resistant Status Classification

Salt resistance was defined as a ≤ 5 mmHg change in mean arterial pressure (MAP) determined between LS and the HS diets. The classification of salt resistance was determined after the participants completed full study protocol. Participants classified as salt sensitive, showing a change in MAP ≥ 5 mmHg from the LS to the HS diet, were excluded from analysis, because they could not be used to test due to BP-independent effects of dietary salt on measured parameters. 

### 2.4. 24-h Urine Samples Analysis and Blood Pressure Measurement 

Urine was collected during the last 24-h period on the LS, HS, and HS+C+E conditions according to the given instructions. Then, the 24-h urine aliquots were analyzed for sodium, potassium, and urea concentration, as well as creatinine coefficient at the Department of Clinical Laboratory Diagnostics, University Hospital Osijek. Daily salt intake based on 24-h urinary sodium excretion was calculated using appropriate formula (1-gram salt (NaCl) = 393.4 mg Na = 17.1 mmol Na). 

BP and heart rate (HR) were measured at the beginning of each visit after a 15 min rest in a seated position using an automated oscillometric sphygmomanometer (OMRON M3, OMRON Healthcare Inc., Osaka, Japan). The final values of BP and HR were the mean of three repeated measurements.

### 2.5. Assessment of Skin Microcirculatory Blood Flow

Laser Doppler flowmetry (LDF) (MoorVMS-LDF, Axminster, UK) was used to assess an overall change in microvascular function by induction of post-occlusive reactive hyperemia (PORH), and to evaluate endothelium-dependent and -independent vasodilation by iontophoresis (noninvasive transdermal application of charged substances) of acetylcholine (ACh) and sodium nitroprusside (SNP), respectively. LDF measurements were performed at each study visit in a temperature-controlled room (mean ± SD temperature = 23.5 ± 0.5°C). Data collection started 30 min after resting in a supine position to acclimatize. The laser Doppler probe was attached to the skin of the volar forearm, 13–15 cm from the wrist, at the same place at each study visit.

The PORH test was performed after a 5-min baseline measurement, by occlusion of the brachial artery that was induced by inflating a pneumatic cuff on the upper arm to 30–50 mmHg above the systolic BP (SBP). Measurements were taken before, during, and after the release of 1-min occlusion. Microcirculatory blood flow was expressed in arbitrary perfusion units and determined by software calculating the area under the curve (AUC) during baseline flow, occlusion, and reperfusion. The result was expressed as the difference between percentage of flow change during reperfusion and occlusion in relation with baseline (R-O% increase). The procedures for the PORH LDF measurements were done according to our previously described protocols [13,28].

Substances for iontophoresis were placed in an iontophoretic drug-delivery electrode that was attached at the site of the LDF probe. After baseline recording for 5 min, either the positively charged vasodilator ACh (1%) was iontophorezed with anodal current applied by means of seven pulses of direct electric current of 0.1 mA for 30 s with 30 s between each dose, or negatively charged SNP (1%) was applied by means of three pulses of 0.1 mA of negative current for 30 s, followed by a four pulses of 0.2 mA for 30 s, with 90 s between each dose. The pulsed iontophoretic protocols are adapted to obtain a stable plateau of the maximal LDF response [14,29]. Microcirculatory blood flow in this test was also expressed in arbitrary perfusion units and determined by software calculating the AUC during baseline flow and during ACh or SNP administration. The result was expressed as a blood flow increase following ACh or SNP administration in relation to baseline flow (ACh or SNP blood flow increase).

### 2.6. Venous Blood Samples Analysis

A venous blood sample was taken after 30 min resting in a supine position at each visit. Blood samples were analyzed for full blood count, plasma electrolytes (sodium, potassium, calcium), urea, creatinine, fasting lipid panels (total cholesterol, high-density lipoprotein cholesterol (HDL), low-density lipoprotein cholesterol (LDL), and triglycerides), fasting blood glucose and high sensitivity C reactive protein (hsCRP) using standard laboratory methods. Plasma renin activity (PRA) and serum aldosterone were measured via commercially available ELISA kits (PRA, No. DB52011, IBL International, Hamburg, Germany; Aldosterone Elisa, No. KAPDB450, DIAsource ImmunoAssays, Louvain-la-Neuve, Belgium). All measurements were performed at the Department of Clinical Laboratory Diagnostics, University Hospital Osijek. 

### 2.7. Measurement of Thiobarbituric Acid Reactive Substances (TBARS) and Ferric-Reducing Ability of Plasma (FRAP)

Venous blood samples for thiobarbituric acid reactive substances (TBARS) and ferric-reducing ability of plasma (FRAP) measurements using spectrophotometry were collected following LS, HS, and HS+C+E conditions in tubes with anticoagulant, snap frozen in liquid nitrogen, and stored in a refrigerator at −80 °C until the experiments were performed. The TBARS method measures the products of lipid peroxidation and presents a measure of oxidative stress level. Since the method is non-specific, because other substances bind to thiobarbituric acid (including proteins), trichloroacetic acid was added to the sample to precipitate the proteins and after that the supernatant used was for further measurement. The absorbance of the sample was measured by Nanophotometer P300 UV/VIS, IMPLEN at 572 and 532 nm with malondialdehyde (MDA) used as a standard (µM MDA) [30]. The FRAP method was used for measuring antioxidant capacity of blood samples. Fe^3+^-TPTZ (2,4,6-tris(2-pyridyl)- s-triazine) is reduced to Fe^2+^-TPTZ in the presence of antioxidants and a blue discoloration occurs. The absorbance of the sample was measured by Nanophotometer P300 UV/ VIS, IMPLEN at 593 nm with as standard (mM/L Trolox) [31]. The general procedures for TBARS and FRAP measurements were done according to previously described protocol in our laboratory [16].

### 2.8. Measurement of Intracellular Reactive Oxygen Species (ROS) Production

The assessment of intracellular ROS production was performed using flow cytometry (FACS Canto II BD Bioscience). Firstly, 2’7’-dichlorodyhydrofluorescein diacetate (DCF-DA) was used to determine the levels of hydrogen peroxide (H_2_O_2_) and peroxynitrate (ONOO-) levels, and dihydroethidium (DHE) was used to determine the level of O_2_∙− in peripheral blood leukocytes (PBLs) (granulocytes, monocytes and lymphocytes). Venous blood samples for the determination of intracellular ROS production in PBLs were collected in tubes containing 6–10% 0.5M ethylenediaminetetraacetic acid (EDTA) anticoagulants. A quantity of 90 µL of whole blood was incubated for 30 min at +4 °C with 10 µL of DCF-DA or DHE (final concentration of 10 µM). After incubation was complete, samples with DCF-DA were washed with 2 mL of pure PBS, resuspended in 450 µL of PBS, and read on a flow cytometer (excitation wavelength 488 nm, 530/30 bandpass filter for analysis). After incubation, 350 μL of PBS was added to the samples with DHE (without rinsing), and the samples were also read on a flow cytometer (excitation wavelength 488 nm, 585/42 bandpass filter for analysis). Data are expressed as median fluorescence intensity. A FACS Canto II flow cytometer (BD Bioscience, Franklin Lakes, NJ, USA) and a Diva 6 program (BD Bioscience, Franklin Lakes, NJ, USA) were used for sample analysis and data storage. Final data analysis was done using the free FSC file analysis software, Flowing software (by Perttu Terho, Cell Imaging Core, Turku Centre for Biotechnology. Turku, Finland). 

### 2.9. Measurement of Serum 8-iso Prostaglandin F2α (8-iso-PGF2a) Protein Concentration

The protein concentration of 8-iso-PGF2ɑ (an isoprostane produced by the non-enzymatic peroxidation of arachidonic acid in membrane phospholipids) were detected using the commercially available ELISA kit (MyBioSource, MBS700957, San Diego, CA, USA). 

### 2.10. Measurement of Serum Cu/Zn Superoxide Dismutase (Cu/Zn SOD), Glutathione Peroxidase 1 (GPx1) and Catalase Protein Concentrations

Serum protein concentrations of Cu/Zn SOD (SOD isoform responsible for the dismutation of O2∙− into H_2_O_2_) (Invitrogen, BMS222, Thermo Fisher Scientific, Waltham, MA, USA, SAD), GPx1 (antioxidative enzyme that catalyze the reduction of H_2_O_2_ to water) (Fine Test, EH0826, Wuhan, Hubei, China) and catalase (antioxidative enzyme that converts H_2_O_2_ to oxygen and water) (Fine Test, EH0643, Wuhan, Hubei, China) were measured via commercially available ELISA kits. All measurements of oxidative stress levels were performed in the Laboratory for Molecular and Clinical Immunology, Department of Physiology and Immunology, Faculty of Medicine University of Osijek.

### 2.11. Measurement of Matrix Metalloproteinase 9 (MMP-9) Protein Concentration

Serum protein concentration of MMP-9 (marker of extracellular matrix remodeling) was measured by commercially available ELISA kit according to the manufacturer recommendations and protocols (eBioscience BMS2016/2, Thermo Fisher Scientific, Waltham, MA, USA). These measurements were performed in the Laboratory for Molecular and Clinical Immunology, Department of Physiology and Immunology, Faculty of Medicine University of Osijek.

### 2.12. Statistical Analysis

All results are reported as the mean ± standard deviation (SD). Two-Way ANOVA and appropriate post hoc tests were used to assess differences between LS and HS diet protocol, and LS and HS+C+E diet protocol within the groups, as well as differences between the HS and HS+C+E group. To assess differences between the HS and HS+C+E groups at baseline, the Student t-test for parametric and the Mann–Whitney test for nonparametric distributions were used. The normality of data distribution was assessed by the Kolmogorov–Smirnov normality test. The correlations between microvascular reactivity (PORH and iontophoresis of ACh) and corresponding variables (salt intake, PRA, serum aldosterone level, FRAP, and TBARS) were determined by Pearson’s or Spearman’s correlation tests when appropriate. A multiple linear regression was used to examine which of the abovementioned variables more significantly predict changes in microvascular function following the HS diet (51 subject, 102 measurements, 5 independent variables). *p* < 0.05 was considered statistically significant. SigmaPlot, version 11.2 (Systat Software, Inc., Chicago, IL, USA) was used for statistical analysis.

## 3. Results

Participants’ anthropometric characteristics are presented in Table 1. All participants were lean and normotensive, had normal full blood counts, renal function, serum electrolytes, fasting blood glucose, hsCRP, and fasting lipid levels. There was no significant difference in all measured parameters at the recruitment in the study (initial measurements) (e.g., age, BMI, waist-to-hip ratio (WHR), BP, HR, and biochemical parameters) between participants who comprised the HS and HS+C+E groups. All participants completed a two-week dietary salt perturbation period; a seven-day LS diet that presented the “wash-out” period (LS), which was followed by a seven-day HS diet without (HS) or with intake of vitamin C and E (HS+C+E). 

### 3.1. Anthropometric, Hemodynamic, and Biochemical Parameters

No significant changes in BMI and WHR where observed after 7 days of HS or HS+C+E diets compared to LS conditions within the groups, just as there were no differences in BMI and WHR between HS and HS+C+E groups (Table 2). 

The values of SBP, DBP, and MAP were similar after HS and HS+C+E diets compared to the LS diet, and between the HS and HS+C+E groups (Table 2). According to changes in MAP after HS or HS+C+E diets compared to LS conditions within the groups (see Method section), all participants were characterized as salt-resistant, since large increases in salt intake across seven days was not accompanied with concomitant increases in BP values. Thus, all the observed changes that followed both HS and HS+C+E diet protocol can be considered as independent of BP.

There were no significant differences in serum sodium, serum potassium, or hsCRP concentrations after HS or HS+C+E diet compared to LS conditions within the groups, nor when these values were compared between HS and HS+C+E groups (Table 2). 

As expected, 24-h urine sodium excretion as well as calculated daily salt intake significantly increased, while PRA and serum aldosterone level significantly decreased following both HS and HS+C+E protocols compared to measurements done after LS diet (Table 2). Values of 24-h sodium excretion, calculated salt intake, PRA, and aldosterone levels following the seven-day diet period did not differ between the HS and HS+C+E groups (Table 2). Seven days of HS or HS+C+E diets did not significantly affect 24-h urinary total volume, creatinine coefficient, urea and potassium excretion compared to the LS diet, just as these parameters did not differ between the HS and HS+C+E groups (Table 2).

### 3.2. Post-Occlusive Reactive Hyperemia, Acetylcholine-Induced Dilation, and Sodium Nitroprusside-Induced Dilation of Forearm Skin Microcirculation

**Figure 1:**
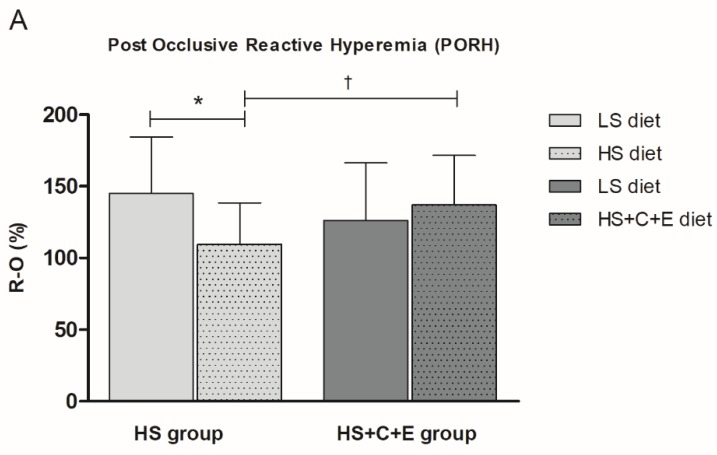

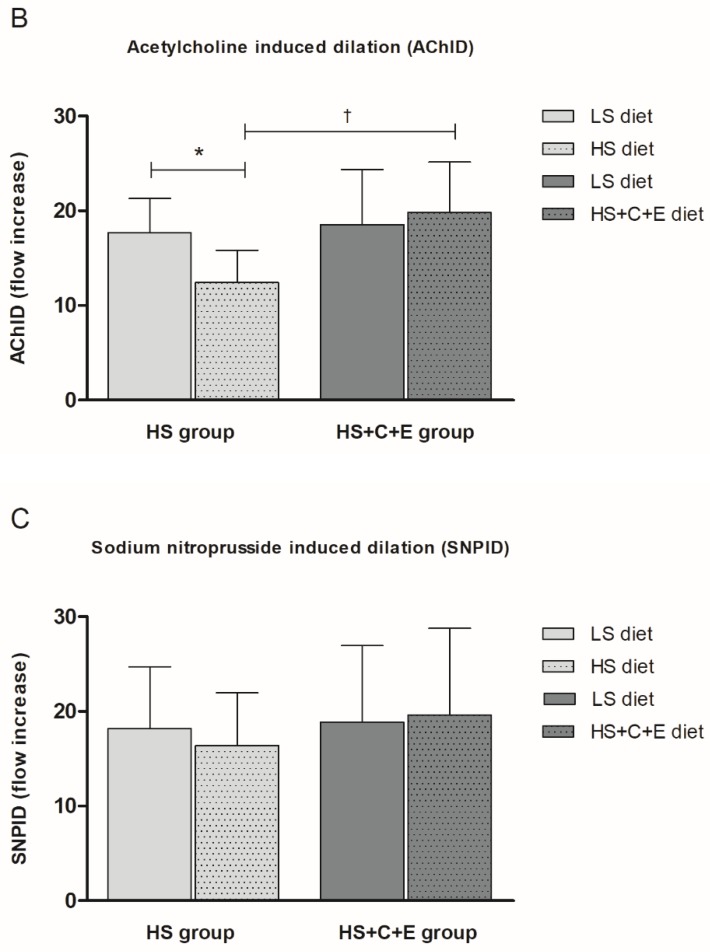
The effect of the 7-day high-salt (HS) diet without (HS group) and with vitamin C and E (HS+C+E group) supplementation on skin microvascular reactivity in young healthy individuals. (**A**) Post-occlusive reactive hyperemia (PORH), (**B**) Acetylcholine-induced dilation (AChID), and (**C**) Sodium nitroprusside induced dilation (SNPID). PORH measurement is expressed as the difference between percentage of flow change during reperfusion and occlusion in relation with baseline (R-O%). AChID and SNPID are expressed as flow increase following ACh or SNP administration compared to baseline flow. Data are presented as average ± SD. LS- low-salt; HS- high-salt; HS+C+E- high-salt + C vitamin + E vitamin; R-O%- change of microvascular blood flow between reperfusion and occlusion (in relation to baseline); AChID- acetylcholine induced dilation; SNPID- sodium nitroprusside induced dilation. *p < 0.05, LS diet vs. HS diet within the HS group. † *p* < 0.05 HS diet vs. HS+C+E diet.

The HS diet significantly decreased both PORH (Figure 1A) and ACh-induced dilation (AChID) (Figure 1B) of forearm skin microcirculation compared to the LS diet within the HS group. Contrastingly, the addition of vitamin C and E to the seven-day HS diet protocol in the HS+C+E group prevented decreases of PORH (Figure 1A) and AChID (Figure 1B) of skin microcirculation compared to values within the LS diet group. Addition of vitamins C and E restored both PORH and AChID in the HS+C+E group compared to the HS group (Figure 1A,B). SNP-induced dilation was similar between LS and HS and HS+C+E diet treatments within groups (Figure 1C), and similar between HS and HS+C+E treatments (Figure 1C).

### 3.3. Markers of Oxidative Stress and Antioxidative Defense

The seven-day HS diet significantly decreased FRAP levels (marker of antioxidative defense) and increased TBARS levels (marker of lipid peroxidation) in the HS group compared to LS conditions, while both FRAP and TBARS remain unchanged after seven-day diet protocol in the HS+C+E group compared to LS conditions within the HS+C+E group (Table 3). The level of FRAP was significantly higher and the level of TBARS was significantly lower in the HS+C+E group compared to the HS group (Table 3).

Similarly, serum protein concentrations of 8-iso-PGF2α (marker of oxidative stress) were significantly increased in the HS group after the HS diet compared to LS conditions, while the addition of vitamin C and E prevented the increase in 8-iso-PGF2α within the HS+C+E group (Table 3). In addition, serum protein concentrations of 8-iso-PGF2α were significantly lower in the HS+C+E compared to the HS groups (Table 3).

Serum concentrations of Cu/Zn SOD and GPx1 proteins (antioxidative enzymes) were not significantly changed in HS or HS+C+E diets compared to the LS diet in respective groups, just as there were no differences in Cu/Zn SOD and GPx1 protein concentrations between the HS and HS+C+E groups (Table 3). While the seven-day HS diet significantly decreased catalase protein concentrations in the HS group compared to the LS diet, there was no statistical significant change in catalase protein concentration following seven days of the HS+C+E diet (HS+C+E group) compared to the respective LS diet period (Table 3). The differences in catalase protein concentrations following correspondent diet protocols were not significant between HS and HS+C+E groups (Table 3).

**Figure 2:**
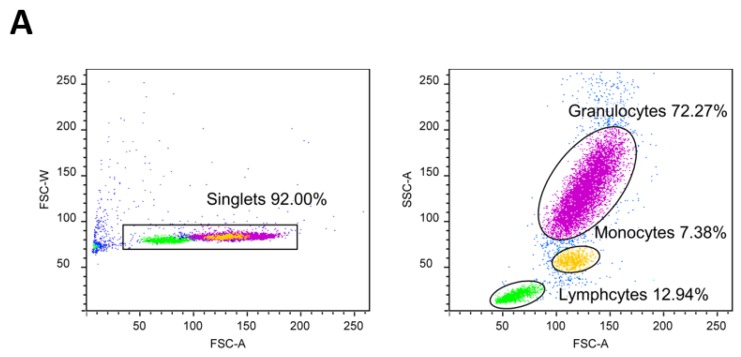

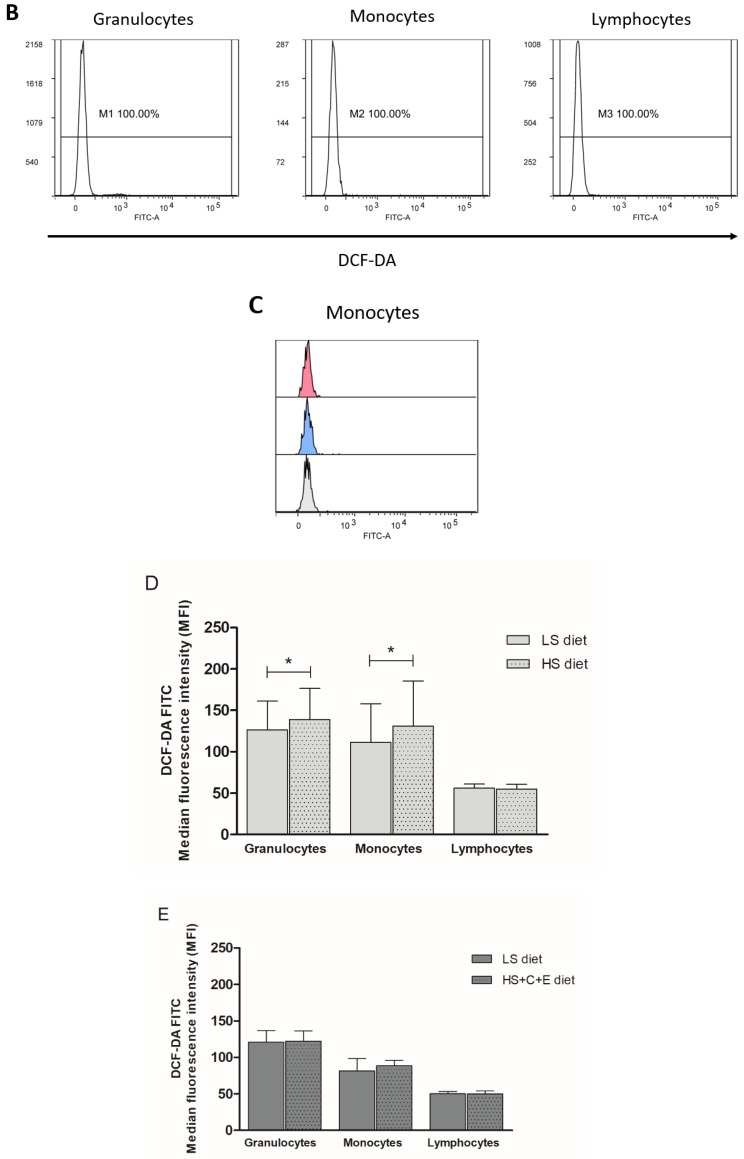
The effect of the 7-day high-salt (HS) diet without (HS group) and with vitamin C and E (HS+C+E group) supplementation on hydrogen peroxide and peroxynitrite (DCF-DA) formation in peripheral blood leukocytes (PBL)—granulocytes, monocytes, and lymphocytes in young healthy individuals. (**A**) shows gating strategy for excluding doublets and defining leukocytes subpopulations based on the size (FSC) and complexity (SSC), (**B**) shows histograms expressing the level of oxidative stress in individual leukocyte populations, and (**C**) shows the comparison of the expression of oxidative stress in monocytes at study enrollment (gray), after 7 days of low-salt (blue) and after 7 days of high-salt diets (red). (**D**) shows median fluorescence intensity of DCF-DA FITC. (**D**,**E**) shows the median fluorescence intensity of DCF-DA FITC on PBLs in HS and HS+C+E groups. Data are presented as mean ± SD. * *p* < 0.05, LS diet vs. HS diet within the HS group.

While the seven-day HS diet protocol significantly increased the median fluorescence intensity of DCF-DA (marker of intracellular production of H_2_O_2_ and ONOO-) in peripheral blood granulocytes and monocytes, but not in lymphocytes of HS group compared to LS diet, it remained unchanged in all three peripheral blood cell lines after a seven-day protocol in the HS+C+E group compared to respective LS diet (Figure 2). The level of DCF-DA in peripheral blood granulocytes, monocytes, and lymphocytes was significantly lower in the HS+C+E group compared to the HS group (Figure 2).

**Figure 3:**
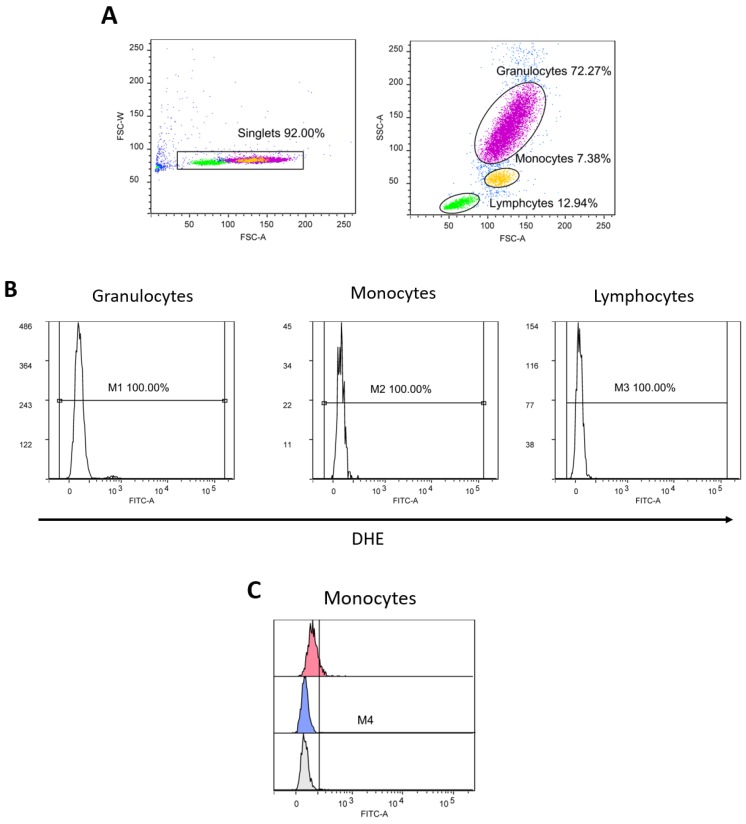

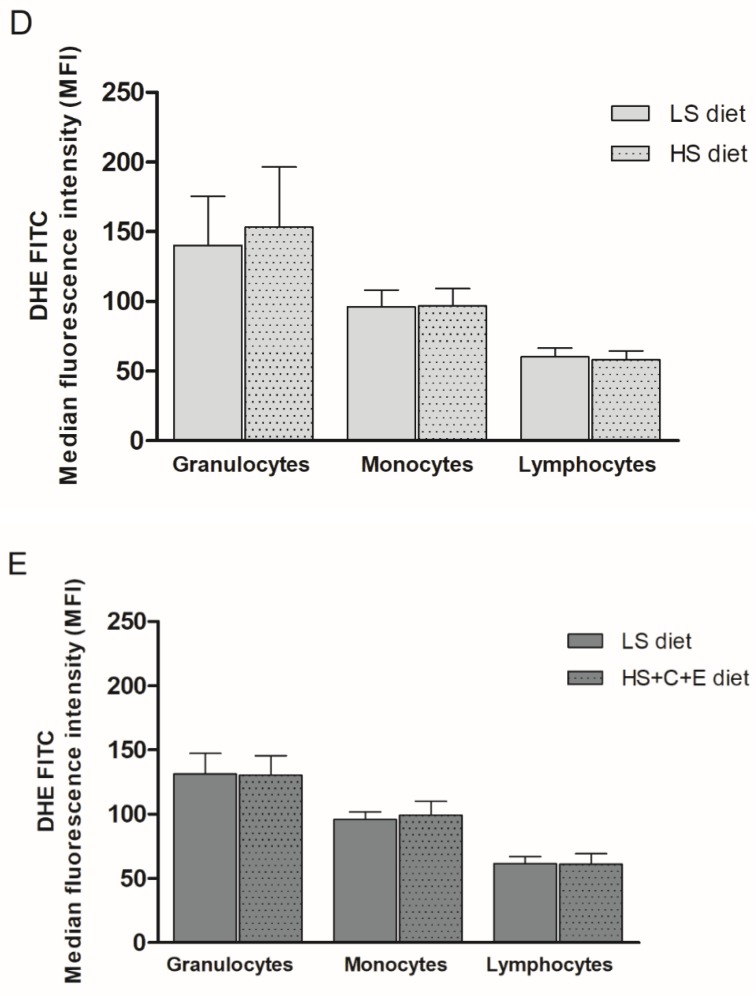
The effect of the 7-day high-salt (HS) diet without (HS group) and with vitamin C and E (HS+C+E group) supplementation on superoxide (DHE) formation in peripheral blood leukocytes (PBL)—granulocytes, monocytes, and lymphocytes in young healthy individuals. (**A**) shows gating strategy for excluding doublets and defining leukocytes subpopulations based on the size (FSC) and complexity (SSC), (**B**) shows histograms expressing the level of oxidative stress in individual leukocyte populations, and (**C**) shows the comparison of the expression of oxidative stress in monocytes at study enrollment (gray), after 7 days of low-salt (blue) and after 7 days of high-salt diets (red). (**D**,**E**) shows the median fluorescence intensity of DHE FITC on peripheral blood leukocytes in HS and HS+C+E groups. Data are presented as mean ± SD.

Median fluorescence intensity of DHE (marker of intracellular production of O2∙-) in peripheral blood granulocytes, monocytes, and lymphocytes did not change significantly after the HS or HS+C+E diet protocols compared to respective LS diet period, just as there was no difference in median fluorescence intensity of DHE in any of blood cell lines between HS and HS+C+E groups (Figure 3).

### 3.4. Serum Protein Concentration of Matrix Metalloproteinase 9 (MMP-9)

Serum protein concentrations of MMP-9 were not significantly different following both the HS diet (MMP-9 ng/mL LS 691.6±162.7 vs. HS 711.2 ± 117.4, *p* > 0.05) or the HS+C+E diet (MMP-9 ng/mL LS 744.4 ± 87.5 vs. HS+C+E 725.1 ± 110.7, *p* > 0.05) compared to the respective LS diet period, just as there was no difference in MMP-9 protein concentrations between the HS and HS+C+E groups (MMP-9 ng/mL HS 711.2 ± 117.4 vs. r HS+C+E 725.1 ± 110.7, *p* > 0.05).

### 3.5. Correlation Between Microvascular Reactivity and Salt Intake/RAS Suppression/Oxidative Stress and Antioxidative Defense Following the High-Salt Diet

As expected, there was a significant weak negative correlation between salt intake and PRA (*r* = −0.389, *p* = 0.045), and significant moderate negative correlation between salt intake and serum aldosterone level (*r* = −0.570, *p* = 0.005). There was no significant direct correlation between RAS components (PRA and serum aldosterone level) and markers of oxidative stress (FRAP, TBARS, or 8-iso-PGF2α). 

A significant weak negative correlation was observed between PORH and 24-h sodium excretion in young healthy participants (*r* = −0.318, *p* = 0.046). Moreover, there was a significant weak positive correlation between PORH and serum aldosterone level (*r* = 0.410, *p* = 0.042), and between PORH and FRAP level (*r* = 0.348, *p* = 0.020). There was no significant correlation between PORH and PRA (*r* = 0.342, *p* = 0.069), and between PORH and TBARS (*r* = −0.056, *p* = 0.715). However, when using a multiple linear regression model to analyze which of the abovementioned parameters (PRA, serum aldosterone level, 24 h sodium excretion, FRAP, and TBARS) most strongly predict the PORH value, the results showed that FRAP and PRA together are strongly positively associated with PORH (*r^2^* = 0.769, *p* = 0.007), rather than FRAP alone (*r^2^* = 0.580, *p* = 0.030). 

There was a significant moderate negative correlation between AChID and 24-h sodium excretion (*r* = −0.508, *p* = 0.002), and a weak but significant positive correlation between AChID and FRAP (*r* = 0.388, *p* = 0.015), as well as a weak but significant negative correlation between AChID and TBARS (*r* = −0.419, *p* = 0.008). There was no significant correlation between AChID and PRA (*r* = −0.113, *p* = 0.586), and between AChID and serum aldosterone levels (*r* = 0.067, *p* = 0.767). A multiple linear regression analysis showed that FRAP (among PRA, serum aldosterone levels, 24 h sodium excretion, FRAP, and TBARS) is a parameter that most strongly predicts the AChID value (*r^2^* = 0.582, *p* = 0.004).

## 4. Discussion

This is the first in vivo study which investigated the mechanisms by which seven-day HS loading affects microvascular endothelial function in young healthy salt-resistant individuals. In particular, the novelty and contribution of this study is that it studied oxidative stress as a potential link between HS intake and endothelial damage, by direct measurement of various markers of oxidative stress and antioxidant defenses, and changes in biochemical markers were confirmed by functional vascular measurements. The novel findings of the present study are twofold. This study has primarily shown that a seven-day HS diet leads to the decrease of antioxidant capacity and increase of oxidative stress independently of BP in young healthy individuals. Second, enhanced antioxidative defense by vitamin C and vitamin E administration during 7 days of HS loading reverses these changes in oxidative status, and prevents impairment of forearm skin microvascular reactivity (PORH and AChID) that were observed with HS dietary intake. Thus, our study suggests the pivotal role of oxidative balance in maintaining normal vascular function during HS loading, independently of BP values. Likewise, the present study has demonstrated that a seven-day HS diet did not cause changes in MMP-9 serum protein concentrations, suggesting that functional vascular alterations, manifested as impaired microvascular reactivity, occur earlier than potential structural remodeling of blood vessels.

### 4.1. High-Salt Diet Impairs Microvascular Function 

An increasing body of evidence indicates the importance of microvascular impairment in the development of CV diseases [32,33,34]. In order to separate well established effect of elevated BP on vascular (endothelial) function from the effect of salt loading, studies were shifted from investigating hypertensive patients to the population of healthy individuals. One of the pioneer studies in the field reported that four weeks of an HS diet (250 mmol Na+/day) did not affect forearm blood flow after metacholine, but enhanced blood flow after SNP administration measured by strain-gauge plethysmography, suggesting that endothelial NO synthesis and release might have been lower after the HS diet [35]. Tzemos et al. reported functional impairment of forearm microvascular endothelial-dependent vasodilation (measured by venous occlusion plethysmography) in healthy individuals following a 5-day HS diet (200 mmol Na+/day) [36]. However, since the HS diet was accompanied by a small but significant increase in BP, the effect of HS intake on endothelial function could not be completely uncoupled from the effect of elevated BP [36]. Later studies have unequivocally shown that a short-term (seven-day) HS diet causes microvascular reactivity impairment in healthy individuals independently of BP changes by decreasing microvascular reactivity in response to local heating (NO-mediated dilation) [15], vascular occlusion (PORH; general microvascular reactivity) [13], and iontophoresis of ACh (ACHID; endothelium-dependent vasodilation) [14]. In addition to impaired NO-mediated response of forearm microcirculation with the HS diet [15], our research group revealed the importance of vasoconstrictor metabolites of cyclooxygenase (COX) enzymes in the impairment of microvascular PORH in HS diets in healthy individuals [13]. Vasodilation of arterioles isolated from human gluteal subcutaneous fat (in vitro) in response to ACh and changes in flow following the seven-day HS diet (250 mmol Na+/day) were no longer NO-dependent, but COX and cytochrome P450 (CYP450) vasodilator mediators took part in this vasodilation [8]. The novelty of the present study is that impaired endothelium-dependent (PORH and AChID), but not endothelium-independent (SNP-induced dilation, SNPID), microvascular vasodilation in forearm skin microcirculation of healthy individuals by HS diet is related to increased oxidative stress and decreased antioxidative defense capacity, independently of changes in BP. 

### 4.2. High-Salt Diet and Vascular Structural Remodeling 

It is well known that the onset of hypertension is related to MMP-9 activation concurrent with a rise in vessel distensibility. The role of MMP-9 in an early stage of endothelial dysfunction is still intriguing. Flamant et al. have detected the increase of MMP activity fed with 5% NaCl for 10 days, which, in early beginning, has a beneficial effect, while later mediates extracellular matrix protein turnover and finally results in vessel rigidification [25]. Furthermore, MMP-9 inhibition attenuates hypertensive cerebrovascular dysfunction in Dahl-salt-sensitive rats fed with 4% NaCl diet during six weeks [37]. Nevertheless, our study has shown no change in MMP-9 activity following the seven-day HS diet, which may suggest that seven days of HS intake is not long enough to change the MMP-9 activity and start vascular remodeling.

### 4.3. High-Salt Diet Increases Oxidative Stress due to a Decrease of Antioxidative Capacity

**Figure 4:**
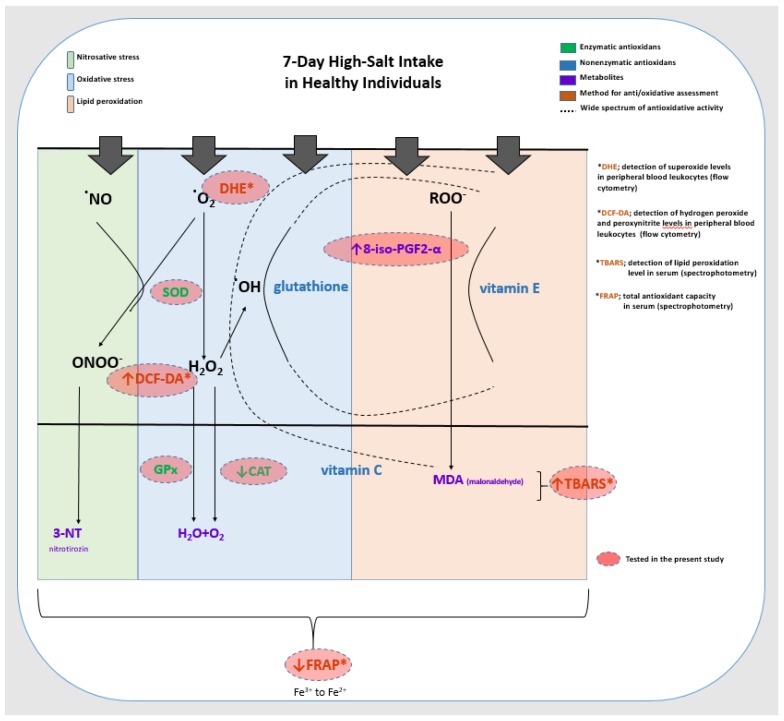
Summary of the effect of the 7-day high-salt (HS) diet on parameters of oxidative stress measured in the present study in young healthy individuals. This figure summarizes the most commonly known effects of the superoxide radical (O_2_•), nitric oxide radical (NO•), hydroxyl radical (OH•), and peroxyl radical (ROO•) in the formation of oxidative stress (blue), nitrosative stress (green), and lipid peroxidation (orange). The effects of enzymatic (green) and non-enzymatic (blue) components of antioxidative defense are also described, as well as the spectrum of their antioxidative activity (full arrow—the reaction they catalyze, dashed line—the spectrum of antioxidative activity). Radicals, metabolites, and enzymes that were measured in the present study as markers of oxidative stress and antioxidative defense are indicated in red filled circles. Seven days of the HS diet increased oxidative stress levels, which manifested as increased lipid peroxidation (increased serum TBARS and 8-iso-PGF2ɑ) and increased intracellular H_2_O_2_ and ONOO- production in peripheral blood leukocytes (increased DCF-DA in granulocytes and monocytes), and decreased antioxidant defense which manifested as decreased antioxidant capacity of plasma (FRAP) and serum catalase protein concentrations in young healthy individuals. ONOO—peroxynitrite; NO•—nitric oxide radical; O_2_•—superoxide; H_2_O_2_—hydrogen peroxide; HO•—hydroxyl radical; ROO•—peroxyl radical; DHE —dihydroethidium; DCF-DA—2’7’-dichlorodyhydrofluorescein diacetate; TBARS—thiobarbituric acid reactive substances; FRAP—ferric-reducing ability of plasma; SOD—superoxide dismutase; CAT—catalase; GPx—glutathione peroxidase.

Increased oxidative stress has been the culprit of impaired vascular reactivity in the HS diet, mainly leading to a decrease in NO bioavailability and partly affecting the production of vasoactive metabolites of arachidonic acid [38,39,40,41,42]. Studies in experimental animal models demonstrated accumulation of O2∙− in vascular walls in various vascular beds (skeletal muscle, cerebral and mesenteric, aorta) following an HS diet [18,19,43]. In addition, treatment with SOD or SOD mimetics reverses ROS accumulation and restores NO levels, as well as endothelium-dependent vasodilation, in animals fed HS diets, to the levels measured in animals fed normal salt diet [17,18,43]. Impairment of endothelium-dependent vasodilation caused by increased O_2_∙− production was diminished by NAD(P)H and xanthine oxidase inhibition in HS fed rats, indicating that the largest sources of O_2_∙− during the HS diet were NAD(P)H oxidase and xanthine oxidase [43]. However, there may be differences among vascular beds and species in the enzymes responsible for increased O2∙− production [39]. Short-term HS loading in experimental animals may contribute to increased ROS (e.g., O_2_∙− level) by disturbing the activity of antioxidative defense mechanisms itself (reducing expression and activity of antioxidant enzymes), or together with increasing activity of pro-oxidant enzymes [16,19,39,44,45]. HS diet can lead to reduced activity of SOD (both Cu/Zn SOD and manganese-SOD) in cerebral resistance vessels [44,45]; however, such effect was not uniform in different vascular beds (e.g., mesenteric resistance arteries, skeletal muscle arterioles) [39]. The results of our earlier study in Sprague–Dawley rats demonstrated that the seven-day HS diet increased oxidative stress levels (O2∙− scavenger prevented impairment of middle cerebral artery reactivity, increased TBARS), and although the activity of antioxidant enzymes (catalase, GPx and SOD) was not changed, the mRNA expression of iNOS and antioxidative enzyme GPx4 in the middle cerebral artery were decreased [16]. Increased ROS may react with other cellular components, like nucleic acids, proteins, and lipids, and thereby may change cellular biochemical and physical properties [46]. Non-enzymatic ROS-induced peroxidation of unsaturated fatty acids, like arachidonic acid, results in the formation of bioactive molecules isoprostanes, e.g., 8-iso-PGF2α, which have an important role in modification of platelet aggregation and vascular tone via activation of the prostanoid TXA2 receptor (TP) [46,47]. In the present study, the seven-day HS diet increased lipid peroxidation (increased TBARS) and the consequent production of 8-iso-PGF2α isoprostane in healthy individuals, which could potentially contribute to microvascular reactivity impairment. Furthermore, the present study tended to translate the experimental model (and potentially results) generated in animal models to healthy human individuals. Hereby, we demonstrated for the first time, that a seven-day HS diet increased oxidative stress levels (increased TBARS, 8-iso-PGF2α and DCF-DA values in peripheral blood leukocytes), and decreased antioxidative defense (reduced FRAP and catalase protein concentration) in young healthy individuals, which is summarized in Figure 4. In addition, the described changes in oxidative status were accompanied by alterations in forearm skin microvascular reactivity, demonstrated as reduced PORH and AChID. These results are in the line with the above discussed results from studies conducted on experimental animals, indicating the pivotal role of oxidative stress among the potential mechanisms mediating HS diet-induced impairment of microvascular function. Interestingly, while studies in experimental animals revealed O2∙− as a major source of oxidative stress during HS loading (e.g., in blood vessel wall), results of the present study demonstrated increased lipid peroxidation and the consequent production of isoprostanes, as well as intracellular production of H_2_O_2_ and ONOO- in PBLs (e.g., granulocytes and monocytes), which could be potentially associated with decreased serum catalase protein concentrations.

### 4.4. Increase in Antioxidative Capacity by Vitamin C and E Peroral Administration Restores Microvascular Reactivity in Healthy Individuals

To confirm the role of oxidative stress in microvascular functional impairment which occurs with HS diets, participants of the present study received peroral vitamin C and vitamin E. Vitamin C is well known for its antioxidant role [48]. It enters endothelial cells, where it can donate electrons to a variety of radicals, with O2∙− being one of the most important, or it can be reduced by the activation of cell surface receptors, such as those for thrombin or advanced glycation end-products [48]. Then, it becomes an ascorbate radical, after which it converts back to ascorbate by NADH and NADPH-dependent reductases. Vitamin E also has a beneficial effect on oxidative status by diminishing lipid peroxidation [49]. In the present study, supplementation of these non-specific ROS scavengers during HS loading restored oxidative status and microvascular reactivity in healthy individuals, suggesting that enhancing the antioxidant defense may have an important role in the prevention of early stages of microvascular impairment caused by oxidative stress due to HS loading. Previously, the beneficial effect of the local infusion of vitamin C on forearm skin vasodilation was reported in patients with chronic kidney disease [50], and in other CV patients, such as improved brachial artery FMD in hemodialysis patients [51]. However, this effect was not well investigated in healthy individuals on HS diets. The present study is the first to confirm the role of oxidative stress in microvascular impairment by showing that enhanced antioxidative defense by everyday vitamin C and vitamin E via oral consumption during an HS diet could prevent the microvascular impairment caused by seven-day HS intake.

### 4.5. The Relationship between the Renin–Angiotensin System, Oxidative Stress and Microvascular Function

Numerous previous studies in genetic animal models strongly support the pivotal permissive role of the RAS in maintaining vascular dilation mechanisms via modulating systemic and cellular oxidative stress levels [12,16,19,41,42,44]. It was demonstrated that infusion of a suppressor dose of ANG II during the period of HS intake prevented the reduction of the expression of antioxidant enzymes (Cu/Zn SOD and Mn SOD) in cerebral circulation, and restored the vasodilator response to ACh in isolated middle cerebral arteries, which were present after HS loading in HS-fed rats [44]. The correlations and multiple linear regression analysis performed in the present study emphasize the importance of early stage oxidative status changes due to RAS suppression in microvascular alterations. FRAP as marker of antioxidant capacity has shown to be statistically the most powerful predictor of microvascular endothelium-dependent vasodilation (AChID) in healthy individuals. More importantly, microvascular reactivity in response to vascular occlusion (PORH) was strongly predicted by both FRAP and RAS activity, rather than FRAP alone. Beside the evident role of oxidative balance, these results once more potentiate the central role of normal RAS function in maintaining vascular homeostasis, which was discussed in our recent paper [14]. 

In conclusion, the present study has demonstrated the substantial role of oxidative imbalance in the impairment of microvascular vasodilation caused by seven-day HS loading, independently of BP levels. Such functional vascular alterations were not accompanied by extracellular matrix remodeling yet, possibly due to the short duration of dietary protocol. Most importantly, functional impairment was restored to normal by peroral supplementation of the antioxidants (vitamin C and E), which opens a new line of investigation in humans, taking into account a wide consumption of these vitamins in the world [22,23,24].

## Figures and Tables

**Table 1:** Initial anthropometric characteristics of the study population at the beginning of the study protocol (baseline).

Parameter	HS Group	HS+C+E Group
N (W/M)	24 (11/13)	27 (15/12)
Age (years)	20 ± 2	20 ± 2
BMI (kg/m^2^)	23.0 ± 3.4	23.3 ± 2.5
WHR	0.78 ± 0.06	0.81 ± 0.05
SBP (mmHg)	119 ± 12	117 ± 12
DBP (mmHg)	73 ± 7	73 ± 10
MAP (mmHg)	88 ± 8	88 ± 9
HR (beats per minute)	75 ± 10	77 ± 11
erythrocytes (× 10e^12^/L)	4.9 ± 0.7	4.8 ± 0.3
hemoglobin (g/L)	145 ± 21	142 ± 12
hematocrit (%)	42.96 ± 5.72	41.31 ± 2.51
leukocytes (× 10e^9^/L)	5.8 ± 0.9	6.1 ± 1.6
thrombocytes (× 10e^9^/L)	224 ± 40	279 ± 66
urea (mmol/L)	4.7 ± 1.7	4.7 ± 1.5
creatinine (µmol/L)	67 ± 15	71 ± 14
sodium (mmol/L)	138 ± 4	139 ± 2
potassium (mmol/L)	4.2 ± 0.1	4.1 ± 0.3
glucose (mmol/L)	5.0 ± 0.5	4.8 ± 0.7
hsCRP (mg/L)	0.8 ± 0.9	1.9 ± 3.2
cholesterol (mmol/L)	4.6 ± 0.8	4.3 ± 0.7
triglycerides (mmol/L)	1.0 ± 0.3	1.1 ± 0.5
HDL cholesterol (mmol/L)	1.5 ± 0.4	1.5 ± 0.4
LDL cholesterol (mmol/L)	2.7 ± 0.7	2.5 ± 0.4

Data are presented as average ± standard deviation (SD). HS—high-salt; HS+C+E—high-salt + vitamin C + vitamin E; N—number of participants; W—women; M—men; BMI—body mass index; WHR—waist-to-hip ratio; SBP—systolic blood pressure; DBP—diastolic blood pressure; MAP—mean arterial pressure; HR—heart rate; hsCRP—high-sensitivity C reactive protein; HDL—high-density lipoprotein; LDL—low-density lipoprotein.

**Table 2:** The effect of the 7-day high-salt diet without (HS group) or with vitamin C and E (HS+C+E group) intake on anthropometric, hemodynamic, and biochemical parameters.

Parameter	HS Group		HS+C+E Group	
	LS Diet	HS Diet	LS Diet	HS+C+E Diet
BMI (kg/m^2^)	22.9 ± 3.5	23.0 ± 3.5	23.4 ± 2.4	23.4 ± 2.4
WHR	0.78 ± 0.07	0.78 ± 0.07	0.81 ± 0.05	0.81 ± 0.06
SBP (mmHg)	117 ± 14	115 ± 15	116 ± 10	116 ± 11
DBP (mmHg)	71 ± 8	71 ± 7	71 ± 8	72 ± 9
MAP (mmHg)	86 ± 9	86 ± 9	86 ± 6	87 ± 8
HR (beats per minute)	73 ± 10	75 ± 10	73 ± 11	77 ± 9
sodium (mmol/L)	137 ± 3	138 ± 2	137 ± 3	138 ± 2
potassium (mmol/L)	4.2 ± 0.3	4.3 ± 0.4	4.2 ± 0.3	4.1 ± 0.2
hsCRP (mg/L)	0.6 ± 0.4	0.6 ± 0.2	1.0 ± 0.9	1.2 ± 1.9
PRA (ng/mL/h)	5.71 ± 3.29	1.83 ± 1.0 *	6.72 ± 4.40	2.85 ± 4.12 *
aldosterone (pg/mL)	169 ± 88	98 ± 64 *	214 ± 76	152 ± 78 *
24 h urine volume (mL)	1351 ± 428	1491 ± 648	1147 ± 485	1186 ± 521
24 h creatinine coefficient (µmol/24h/kg)	198 ± 57	195 ± 58	171 ± 50	163 ± 63
24 h urine urea (mmol/dU)	294 ± 127	298 ± 114	289 ± 98	251 ± 94
24 h sodium (mmol/dU)	118 ± 40	244 ± 97 *	118 ± 50	219 ± 99 *
24 h potassium (mmol/dU)	48 ± 20	50 ± 24	43 ± 14	41 ± 22
calculated salt intake (g/day)	6.9 ± 2.4	14.3 ± 5.7 *	6.9 ± 2.9	13.0 ± 5.6 *

Data are presented as average ± standard deviation (SD). HS—high-salt; LS—low-salt; HS+C+E—high-salt + vitamin C + vitamin E; BMI—body mass index; WHR—waist-to-hip ratio; SBP—systolic blood pressure; DBP—diastolic blood pressure; MAP—mean arterial pressure; HR—heart rate; hsCRP—high-sensitivity C reactive protein; PRA—plasma renin activity. * *p* < 0.05 difference between LS diet and HS diet or LS diet and HS+C+E diet within groups.

**Table 3:** The effect of the 7-day high-salt diet without (HS group) or with vitamin C and E (HS+C+E group) intake on markers of oxidative stress and antioxidative defense.

Parameter	HS Group		HS+C+E Group	
	LS Diet	HS Diet	LS Diet	HS+C+E Diet
FRAP (mM/L TE)	0.45 ± 0.08	0.38 ± 0.08 *	0.48 ± 0.11	0.46 ± 0.11 †
TBARS (µm/MDA)	0.45 ± 0.07	0.60 ± 0.22 *	0.42 ± 0.21	0.42 ± 0.15 †
8-iso-PGF2α (pg/mL)	1232 ± 184	1442 ± 114 *	1131 ± 142	1146 ± 191 †
CuZn SOD (ng/mL)	82.9 ± 34.0	82.8 ± 45.6	97.3 ± 39.8	86.0 ± 38.6
GPx1 (ng/mL)	5.99 ± 3.80	6.29 ± 4.16	4.71 ± 3.19	3.76 ± 2.59
catalase (pg/mL)	347 ± 495	261 ± 462 *	249 ± 375	186 ± 349

Data are presented as average ± standard deviation (SD). HS—high-salt; LS—low-salt; HS+C+E—high-salt + vitamin C + vitamin E; FRAP—ferric reducing ability of plasma; TBARS—thiobarbituric acid reactive substances; 8-iso-PGF2α—8-iso Prostaglandin F2α; CuZn SOD—copper zinc superoxide dismutase; GPx1—glutathione peroxidase 1. * *p* < 0.05 difference between the LS diet and HS diet or LS diet and HS+C+E diet within groups; † *p* < 0.05 difference between HS and HS+C+E groups.
